# Transient C5 Palsy After Full-Endoscopic Posterior Cervical Foraminotomy

**DOI:** 10.1155/cro/7787076

**Published:** 2025-07-02

**Authors:** João Paulo Machado Bergamaschi, Ariel Falbel Lugão, Rangel Roberto de Assis, Kelsen de Oliveira Teixeira, Fernando Flores de Araújo, Thiago Queiroz Soares, Gustavo Vitelli Depieri, Álvaro Dowling, Robson Cruz de Oliveira, Fernanda Wirth, Fábio da Silva Forti, Helton Luiz Aparecido Defino

**Affiliations:** ^1^Atualli Spine Care Clinic, São Paulo, Brazil; ^2^Department of Orthopedics and Anesthesiology, University of São Paulo, Medical School of Ribeirão Preto, Ribeirão Preto, Brazil; ^3^DWS Spine Clinic Center, Santiago, Chile; ^4^Atualli Institute, São Paulo, Brazil

**Keywords:** endoscopic spine, postoperative C5 paralysis, spine, surgery, total endoscopic foraminotomy, transient cervical paralysis

## Abstract

**Introduction:**

Postoperative C5 paralysis is defined as new-onset and/or progressive muscle weakness with mild or no sensory disturbances occurring as a result of probable neuropraxia of the cervical root due to injury during surgery.

**Case Report:**

A 40-year-old female patient underwent endoscopic technique for treatment of cervical foraminal stenosis, level C4–C5. The procedure proceeded without incident in the intraoperative period. In the immediate postoperative period, the patient developed a motor deficit, Grade 2 muscle strength of the right deltoid muscle, and right C5 allodynia. Early and multidisciplinary treatment resulted in complete recovery of the neurological deficit and satisfactory evolution of the presented case.

**Discussion:**

Although postoperative C5 palsy is one of the most common postoperative complications after multilevel cervical decompression surgery, its exact mechanism remains poorly understood. Despite the various studies in this field and the possible causes described, there is still no absolute confirmation, so the formulation of hypotheses depends on clinical monitoring and postoperative examinations. The indicated treatment depends on the causal suspicion and pathophysiology and ranges from clinical drug therapy to physical therapies and/or rehabilitation.

**Conclusion:**

C5 paralysis after endoscopic surgery, although rare, is a potential complication. The likely pathophysiology is multifactorial: anatomic features of C5, manipulation of the root, and use of the bipolar in the foraminal region.

## 1. Introduction

Postoperative C5 palsy is a known complication of cervical decompression surgery and is defined as new and/or progressive muscle weakness with mild or no sensory disturbance that occurs as a result of neuropraxia of the cervical root due to injury during surgery. This palsy is usually associated with a loss or decrease in strength of the deltoid muscle, but may also affect other muscles: supraspinatus, infraspinatus, subscapularis, biceps brachii, brachialis, brachioradialis, teres minor, serratus anterior, levator scapulae, rhomboid minor, and rhomboid major. Patients with C5 palsy are therefore usually unable to lower the shoulder. Some epidemiological studies have investigated C5 palsy after cervical decompression surgery and found a mean incidence of 6.3% for this condition including open and minimally invasive techniques (reported cases ranged from 1% to 29%) [1]. Another Korean study reported an incidence ranging from 0% to 2.61%, with a mean of 0.58% in more than 15,000 patients, that is, it is a rare event [2].

Postoperative C5 palsy may manifest immediately after surgery or within 2 months after surgery but usually occurs in the first week after surgery, and its prognosis is usually good [3]. Because it is a relatively common surgical complication of multilevel cervical decompression that is distressing to both the surgeon and the patient, it has been extensively described and studied in the literature. Authors such as Scoville and Stoops reported neurologic complications after cervical laminectomy and foraminotomy with the conventional open technique as early as 1961 and 1962, respectively [4, 5].

With the advent of minimally invasive techniques, the incidence of these complications has tended to decrease, but they are still a problem. There are still descriptions of neurologic involvement of the C5 root in various surgical modalities, but no reports of this type of injury have been found after full endoscopic decompression of the cervical spine. With the increase and popularization of endoscopic techniques, there has also been growing concern about potential complications and the need to define and improve ways to prevent them and/or treat them appropriately. Taking into account the possible complications and their prevention and treatment, this article reports on a case of C5 paralysis following full-endoscopic foraminotomy of the cervical spine.

## 2. Case Report

**Figure 1:**
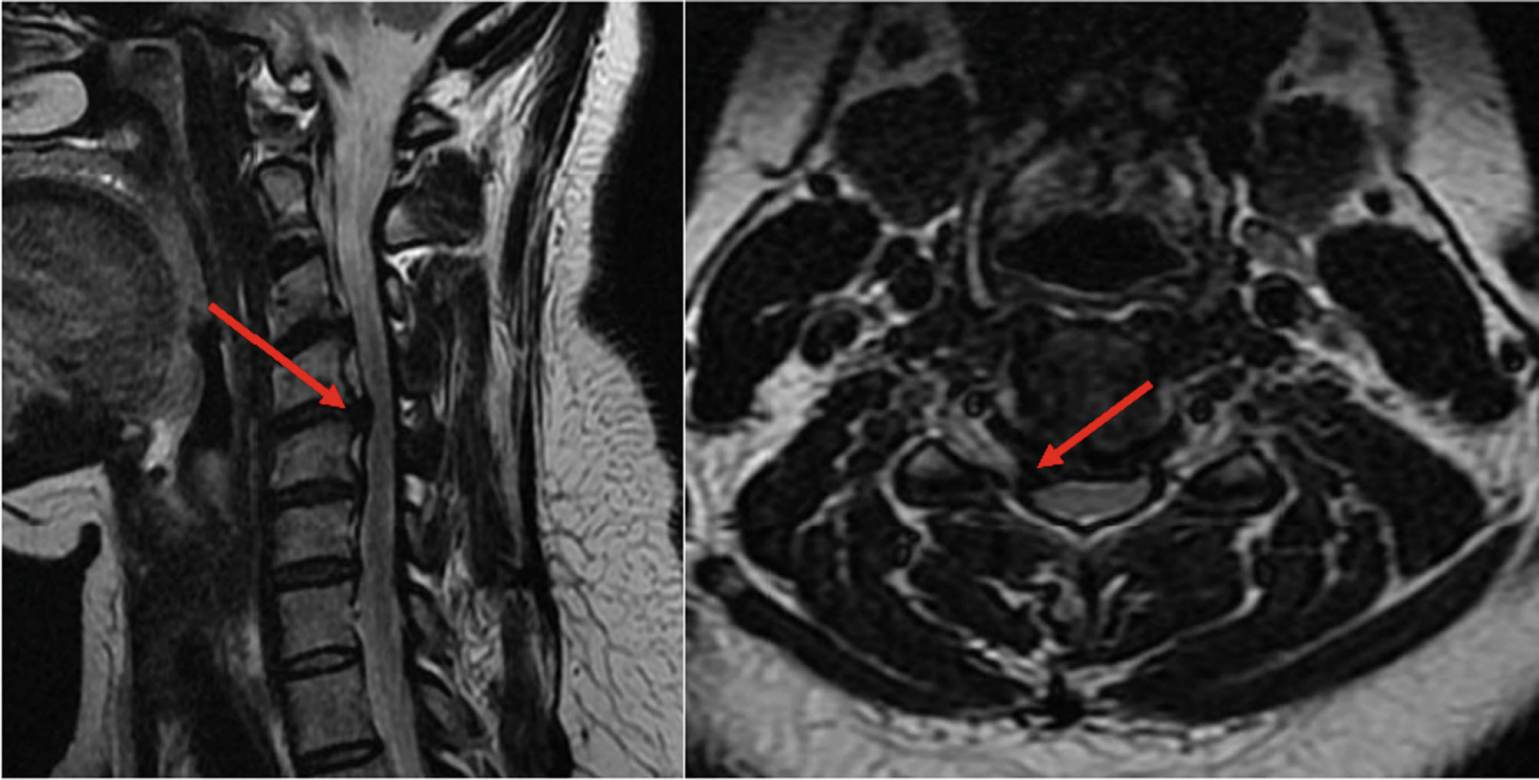
Cervical MRI (sagittal and axial sections) showing C4–C5 disc herniation and right foraminal stenosis.

We present the case of a 40-year-old female patient who had been complaining of neck pain for more than 2 years with a Visual Analogue Pain Scale (VAS) score of 6 and radiation to the right upper extremity (VAS = 7). She had already undergone drug treatment and rehabilitation (physical therapy and acupuncture) without improvement of symptoms. Three months prior to admission, she noted hypoesthesia of C5 and worsening pain. The patient had no motor deficit and normal reflexes. Magnetic resonance imaging (MRI) confirmed the hypothesis of right C5 mild compression due to disc herniation and C4–C5 foraminal stenosis (Figure 1). Dynamic radiographs showed no evidence of instability. Computed tomography (CT) revealed mild C4–C5 facet hypertrophy and showed no calcification of the disc or ligamentum flavum (images from dynamic radiographs and CT not available). At that time, the decision was made to perform a completely endoscopic posterior foraminotomy of the right C4–C5. The patient had no other diagnoses that would have made us think of other problems. A root MRI was not performed at this time.

### 2.1. Surgical Description

**Figure 2:**
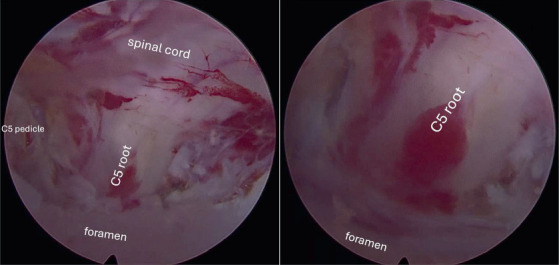
Intraoperative image showing the decompressed C5 root.

• The patient was under general anesthesia and placed in the prone position with slight flexion of the cervical spine.
• The puncture site was confirmed by fluoroscopy in anteroposterior view (lateral line of the cervical interlaminar bone window) and lateral view (parallel to the inclination of the disc to be approached).
• We made a 6-mm transverse incision, opened the fascia, and inserted the dilator and working cannula, always making sure that the bone was in contact with the lateral edge of the blades or the medial edge of the facet joint. The dilator and working cannula were used as an instrument to detach the superior and inferior lamina muscles at the level to be reached.
• The first step of this approach was to determine the V point or Y point, which is the lateral edge of the interlaminar bone window.
• Decompression of the bone was initiated from the superior lamina using a cutting drill. The diameter of the drill was used to calculate the extent of bone resection at the inferior articular process of the parietal vertebra near the facet joint.
• The next step of bone decompression was the resection of the upper part of the lamina of the caudal vertebra.
• Finally, we performed a foraminotomy to achieve lateral decompression up to approximately 50% of the facet joint. At this stage, we aimed for pedicle-to-pedicle (caudal and cranial) decompression, and the most important structure to be partially resected was the superior articular process of the caudal vertebra because it was primarily responsible for foraminal cervical stenosis.
• During foraminotomy, the ligamentum flavum was detached from the bone. After opening the entire ligamentum flavum, more severe bleeding was expected because there is a venous plexus between this structure and the spinal cord, so prophylactic hemostasis with the bipolar is a fundamental step in preventing more severe bleeding.
• The disc was accessed through the axilla of the right C5 root, adjacent to the C5 pedicle.
• With a light cranial manipulation of the root, we removed the herniating fragment with fine forceps.
• Hemostasis was then performed in the foraminal area (Figure 2).
• After removing the working cannula, we routinely infiltrated the incision with ropivacaine.
• Suturing was performed with an intradermal suture using 3-0 Monocryl and a sterile dressing. The procedure was performed without intraoperative interference, with no possible incidental contusion of the root perceived or visualized by the instruments themselves.

**Figure 3:**
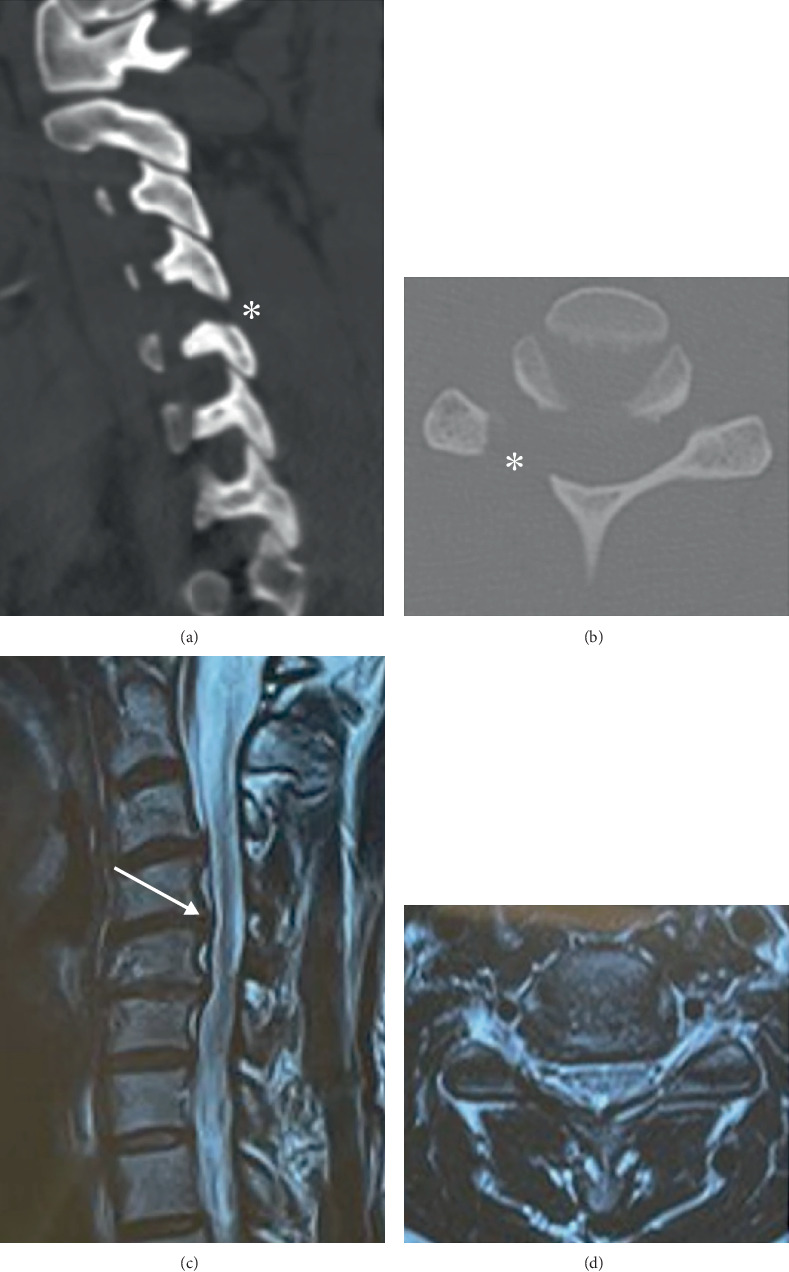
Postoperative exams. (a, b) CT showing satisfactory right C4–C5 foraminotomy (asterisk). (c, d) MRI with reduction of the herniated volume (arrow).

In the immediate postoperative period, despite partial improvement of neck pain and radiating pain (VAS = 4), a Grade II right C5 according to the Manual Muscle Testing (MMT) and allodyne muscle strength in the right deltoid region were observed. Drug treatment (pregabalin, duloxetine, and corticosteroids, in addition to analgesics) and physical rehabilitation (analgesics and motor physiotherapy) were started immediately. Postoperative examinations demonstrated satisfactory foraminotomy and discectomy, with decompression of the C4–C5 level on the right (Figure 3).

**Figure 4:**
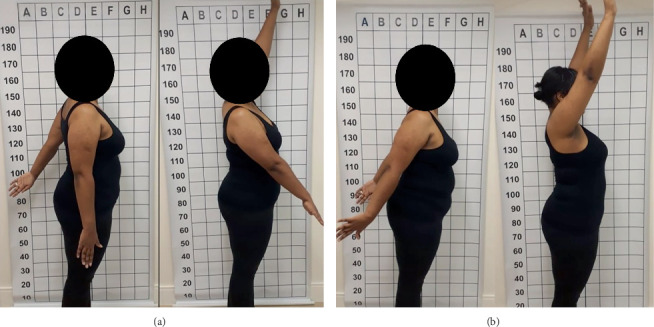
Right upper limb elevation test. (a) Immediate postoperative period, with motor deficit in the right deltoid (muscle strength Grade II). (b) After 4 weeks of intensive rehabilitation and drug treatment (muscle strength Grade V).

After 4 weeks, there was regression of the deficit (Figure 4) and improvement of allodynia, and the medications were gradually discontinued. Two years after surgery, pain scores in the cervical spine and right arm were 1 and 0, respectively.

## 3. Discussion

### 3.1. C5 Palsy

Although postoperative C5 palsy is one of the most common postoperative complications after multilevel neck decompression, the exact mechanism remains poorly understood. For this reason, several studies have investigated and defined some of the risk factors for postoperative C5 palsy, which include age, sex, preexisting anatomic and biomechanical features, instrument use, and number of levels treated [6–9].

A recent systematic review published by Gu et al. analyzed the incidence and risk factors for C5 palsy after posterior cervical decompression and found that the most important risk factors for postoperative C5 palsy were specifically: excessive spinal cord deviation, preexisting intervertebral foraminal stenosis, ossification of the posterior longitudinal ligament, laminectomy, and male sex [10].

### 3.2. Mechanisms and Risk Factors

Although the risk factors seem to be well defined, the exact pathophysiology leading to this problem is still unclear. This debate has been ongoing for more than two decades, and there are numerous plausible etiologies. Sakaura et al. summarized the possible pathological mechanisms of postoperative C5 palsy, including: intraoperative nerve root injury, stretching effect of the nerve root due to posterior spinal cord migration, ischemia of the spinal cord due to decreased blood supply to the radicular arteries, and reperfusion injury of the spinal cord [3].

In the case reported here, other important possibilities include direct manipulation of the root at its origin (preganglionic and ganglionic region) and thermal injury during the procedure. Therefore, when analyzing the procedure performed, we must consider the specific characteristics of the technique, such as the feature of being a minimally invasive procedure through the posterior approach, causing virtually no structural or biomechanical changes to the spine, while specific decompression occurs without exposing large medullary spaces. Therefore, factors such as root overload or medullary displacement seem less likely. In addition, we always preserve at least 50% of the facet joint to avoid possible hypermobility or possible iatrogenic instability, because as discussed by Zdeblick et al. [11], when more than 50% of the facet is resected, torsional stiffness decreases dramatically [11]. On the other hand, because of the small access route and the need to place the instruments directly in the periradicular space, the hypothesis of injury due to manipulation and direct contact with the root or thermal injury is more likely.

### 3.3. Anatomic Features

The fact that the C5 root is highly susceptible to injury may be related to three anatomic features of the cervical spine: (1) the zygoapophyseal joint at C4–C5, which protrudes more anteriorly than the other joints; (2) the roots and root of C5, which are shorter than those of the other segments; and (3) the C5 segment is usually the apex of the decompression area in laminoplasty, and the extent of posterior cord displacement is thought to be greatest at the C5 level [12]. Some anatomic features of the C5 root make it more vulnerable to potential iatrogenic injury during surgery in the region. Because of the lack of clearance, the C5 and C6 nerve roots must be retracted more than other nerve roots to obtain a clear ideal view and access to the disc [13]. In an anatomical study, Hwang et al. showed that the inferior border of the axilla of the C5 root is closer to the inferior border of the disc than the other cervical roots, making access to the disc more difficult [13].

### 3.4. Intraoperative Observations

On the basis of intraoperative observations in laminoplasties, Hirabaiashi et al. was one of the first to suggest that the involvement of the C5 root might be due to the effect of stretching causing residual ossification of the posterior longitudinal ligament in the nerve roots [14]. In this regard, Tsuzuki et al. had already proposed in 1996, based on a pathological analysis, that when perineural fibrosis occurs within a stenosis of the intervertebral foramen and traction is applied to the nerve root, the ventral root becomes more anchored than the dorsal root and suffers a lesion with continuous stretch and motor deficit, whereas the dorsal root suffers a lesion with transient stretch [14, 15].

### 3.5. Radiological Features

Based on radiological studies such as postoperative control MRI, Shiozaki et al. found that there is a common displacement of the spinal cord after decompression surgery. The mean posterior displacement of the spinal cord after cervical laminoplasty was 2.8 mm at 24 h, with a maximum at the C5 level that decreased to 1.9 mm at 2 weeks. In the case of C5 palsy, posterior displacement at 24 h was greatest at C5 in all cases he analyzed. C5 palsy could be avoided if the extent of the dura mater, which correlates strongly with posterior displacement of the spinal cord, could be controlled [16].

### 3.6. Traction of the Radicular Artery

Komagata et al. suggested that traction of the radicular artery after posterior displacement of the spinal cord may decrease blood flow in the spinal cord, resulting in local ischemia. The cervical spinal cord is supplied by the radicular arteries and in the C5 segment is the main source of blood supply, resulting in ischemic root injury. The palsy may be transient and recover functionally after restoration of blood supply if ischemia occurs within a short time. This mechanism predominates in posterior tracts and would rarely occur in anterior decompression procedures [17].

### 3.7. Reperfusion Injury

Reperfusion injury refers to the rapid reperfusion of oxygenated blood into the ischemic tissue, resulting in cellular damage and a paradoxical decrease in blood flow. Intracellular calcium accumulates as a result of the disruption of calcium homeostasis caused by low oxygen partial pressure. Elevated calcium levels convert endothelial xanthine dehydrogenase to xanthine oxidase, a critical enzyme that mediates free radical production during reperfusion [3, 18].

### 3.8. Free Radicals

Most free radicals originate from the conversion of molecular oxygen to superoxide radicals, which directly damage DNA and structural elements of the cell. After cell membrane injury, stimulation of vasoconstrictor prostaglandins such as thromboxane A2 leads to microvasospasm and thrombosis [3, 18].

### 3.9. Diagnosis

C5 palsy immediately after surgery is probably due to direct injury to the nerve root. Iwamoto et al. and Satomi et al. reported that in most patients diagnosed with C5 change in the immediate postoperative period, the loss of neurologic function appeared to be due to direct injury to the nerve structures by surgical cutting and/or grasping instruments. This theory of intraoperative nerve injury cannot explain many cases of C5 palsy, which usually occur several days after surgery [19, 20]. Previous injuries also include thermal injury to the nerve root, either by direct burn with coagulation instruments such as bipolar or by indirect thermal trauma from drills and/or augers [14].

### 3.10. Manipulation and Displacement

Finally, both manipulation and displacement appear to be causal factors along with another anatomic problem. Usami et al. demonstrated that the C5 root has a shorter distance between the branching of the nerve near the dura mater and the beginning of the periradicular fiber sheath, making it more adherent to the posterior longitudinal ligament [21].

### 3.11. Prognosis

In most cases described in a wide variety of techniques, the prognosis of the patient with this type of injury has been quite favorable. A study involving 21 centers and including 15,097 cases of cervical surgery, due to different ethiologies, observed the occurrence of C5 palsy in only 88 cases (0.58%). Only 4 patients did not recover during the postoperative follow-up and more than 50% of them had complete recovery 6 months after surgery [2].

### 3.12. Treatment

Indicated treatment depends on causal suspicion and pathophysiology and ranges from clinical drug therapy (administration of neuroprotective agents such as anticonvulsants, antioxidants, calcium channel blockers, and statins), to physical therapy and/or rehabilitation (with strengthening techniques and/or simply proper positioning: The patient's elbow should be flexed and the shoulder slightly abducted to reduce the distracting force on the C5 nerve root, which can be easily achieved by simply placing a pillow under the armpit while the patient is lying in bed), to a surgical approach with extension of decompression, removal of implants and/or bone fragments or residual soft tissues (if compression and/or indirect stretching is suspected) [18, 22–24].

## 4. Conclusion

C5 palsy after endoscopic surgery, although rare, is a possible complication. The likely pathophysiology is multifactorial: anatomic features of C5, manipulation of the root, and use of the bipolar in the foraminal region. Indicated treatment depends on causal suspicion and pathophysiology and ranges from clinical drug therapy to physical therapy and/or rehabilitation.

Case reports such as this are important, not to limit and/or question the indication for a promising technique, but to alert the spine surgeon to allow for better diagnosis and detailed preoperative case-by-case evaluation and safety measures.

## Data Availability

The data that support the findings of this study are available from the corresponding author upon reasonable request.

## References

[1] Wang T., Wang H., Liu S., Ding W.-Y. (2017). Incidence of C5 Nerve Root Palsy After Cervical Surgery: A Meta-Analysis for Last Decade. *Medicine (Baltimore)*.

[2] Oh J. K., Hong J. T., Kang D. H. (2019). Epidemiology of C5 Palsy After Cervical Spine Surgery: A 21-Center Study. *Neurospine*.

[3] Sakaura H., Hosono N., Mukai Y., Ishii T., Yoshikawa H. (2003). C5 Palsy After Decompression Surgery for Cervical Myelopathy: Review of the Literature. *Spine (Phila pa 1976)*.

[4] Scoville W. B. (1961). Cervical Spondylosis Treated by Bilateral Facetectomy and Laminectomy. *Journal of Neurosurgery*.

[5] Stoops W. L., King R. B. (1962). Neural Complications of Cervical Spondylosis: Their Response to Laminectomy and Foramenotomy. *Journal of Neurosurgery*.

[6] Imagama S., Matsuyama Y., Yukawa Y. (2010). C5 Palsy After Cervical Laminoplasty: A Multicentre Study. *Journal of Bone and Joint Surgery. British Volume (London)*.

[7] Shou F., Li Z., Wang H., Yan C., Liu Q., Xiao C. (2015). Prevalence of C5 Nerve Root Palsy After Cervical Decompressive Surgery: A Meta-Analysis. *European Spine Journal*.

[8] Bydon M., Macki M., Kaloostian P. (2014). Incidence and Prognostic Factors of c5 Palsy. *Neurosurgery*.

[9] Blizzard D. J., Gallizzi M. A., Sheets C. (2015). The Role of Iatrogenic Foraminal Stenosis From Lordotic Correction in the Development of C5 Palsy After Posterior Laminectomy and Fusion. *Journal of Orthopaedic Surgery and Research*.

[10] Gu Y., Cao P., Gao R. (2014). Incidence and Risk Factors of C5 Palsy Following Posterior Cervical Decompression: A Systematic Review. *PLoS One*.

[11] Zdeblick T. A., Zou D., Warden K. E., McCabe R., Kunz D., Vanderby R. (1992). Cervical Stability After Foraminotomy. A Biomechanical In Vitro Analysis. *The Journal of Bone and Joint Surgery. American Volume*.

[12] Tsuzuki N., Tanaka H., Abe R. (1991). Cervical Radiculopathy Occurring After the Posterior Decompression of the Cervical Spinal Cord (in Japanese). *Rinsho-Seikeigeka*.

[13] Hwang J.-C., Bae H.-G., Cho S.-W., Cho S.-J., Park H.-K., Chang J.-C. (2010). Morphometric Study of the Nerve Roots Around the Lateral Mass for Posterior Foraminotomy. *Journal of Korean Neurosurgical Association*.

[14] Hirabayashi K., Toyama Y., Chiba K. (1999). Expansive Laminoplasty for Myelopathy in Ossification of the Longitudinal Ligament. *Clinical Orthopaedics and Related Research*.

[15] Tsuzuki N., Abe R., Saiki K., Iizuka T. (1996). Tension-Band Laminoplasty of the Cervical Spine. *International Orthopaedics*.

[16] Shiozaki T., Otsuka H., Nakata Y. (2009). Spinal Cord Shift on Magnetic Resonance Imaging at 24 Hours After Cervical Laminoplasty. *Spine (Phila pa 1976)*.

[17] Komagata M., Nishiyama M., Endo K., Ikegami H., Tanaka S., Imakiire A. (2004). Prophylaxis of C5 Palsy After Cervical Expansive Laminoplasty by Bilateral Partial Foraminotomy. *The Spine Journal*.

[18] Chiba K., Toyama Y., Matsumoto M., Maruiwa H., Watanabe M., Hirabayashi K. (2002). Segmental Motor Paralysis After Expansive Open-Door Laminoplasty. *Spine (Phila pa 1976)*.

[19] Satomi K., Ogawa J., Ishii Y., Hirabayashi K. (2001). Short-Term Complications and Long-Term Results of Expansive Open-Door Laminoplasty for Cervical Stenotic Myelopathy. *The Spine Journal*.

[20] Iwamoto Y., Fujimura S., Nishi Y. (1996). Neurological Complications in Early Stage After Expansive Open-Door Laminoplasty (in Japanese). *Bessatsu Seikeigeka*.

[21] Usami Y., Yokota A., Kondo Y., Neo M. (2022). Morphology of Cervical Periradicular Fibrous Sheath and Nerve Roots in Relation to Postoperative C5 Palsy. *The Spine Journal*.

[22] Yonenobu K., Hosono N., Iwasaki M., Asano M., Ono K. (1991). Neurologic Complications of Surgery for Cervical Compression Myelopathy. *Spine (Phila pa 1976)*.

[23] Ikenaga M. (2002). A Fifth Cervical Root Paralysis After Corpectomy and Anterior Fusion Over 4 Levels of a Cervical Spine. *Journal of the Japanese Society for Spine Research*.

[24] Yamashita T., Yokogusu K., Yokozawa H. (1996). C5 Nerve Palsy After Cervical Laminoplasty: An Analysis of Three Cases (in Japanese). *Seikei Geka*.

